# *Psoralea diturnerae* and *P. vanberkelae* (Psoraleeae, Fabaceae): two new species restricted to the Core Cape Region of South Africa

**DOI:** 10.3897/phytokeys.44.8999

**Published:** 2015-01-15

**Authors:** Abubakar Bello, Charles H. Stirton, Samson B.M. Chimphango, A. Muthama Muasya

**Affiliations:** 1 Bolus Herbarium, Biological Sciences Department, University of Cape Town, Private Bag X3, Rondebosch 7700, South Africa

**Keywords:** Fabaceae, Leguminosae, New species, Endemic, *Psoralea*, Psoraleeae, South Africa, Taxonomy

## Abstract

Two new species of *Psoralea* L. are described: *Psoralea diturnerae* A. Bello, C.H. Stirt. & Muasya, **sp. nov.** and *Psoralea vanberkelae* C.H. Stirt., A. Bello & Muasya, **sp. nov.** *Psoralea diturnerae* is endemic to the Outeniqua mountains (Camferskloof) and is characterised by a mass of numerous basal shoots out of which emerge 2–3 woody stems up to 2 m tall, 3-foliolate needle-like leaflets at the base of the seasonally growing shoot reducing to one towards the apex and bearing numerous 1–3-flowered axillary inflorescences along its length; each mauve to purple and white flower subtended by a trifid cupulum. *Psoralea vanberkelae* is characterised by its spreading mounding habit, short tightly packed fleshy leaves, with large impressed papillae, densely glandular short broadly triangular stipules, pale to intense mauve to deep blue flowers, standard with a dark purple central blotch above a M-shaped white patch situated above claw, and khaki seeds with purple flecks.

## Introduction

The predominantly southern African genus *Psoralea* L. is a young lineage (ca. 2 million years old, ± 75 species) which has diversified rapidly within the Fynbos Biome and related habitats in South Africa (Dludlu et al. 2013). New species in this genus are discovered regularly as remote areas are being explored (Stirton et al. 2011, 2012) and many species remain undescribed (Stirton and Schutte 2012). The genus is commonly found in mountain fynbos along drainage systems (river beds, stream banks, seepage areas), occurring frequently on sandstone derived soils across the Greater Cape Floristic Region (Stirton and Schutte 2000, 2012). However, there are a number of species, marginal to the main generic distribution, that have adapted to surviving in drier conditions along the arid Fynbos-Succulent Karoo boundary (e.g. *Psoralea angustifolia* Jacq., *Psoralea glaucescens* Eckl. & Zeyh., *Psoralea karooensis* C.H. Stirt., Muasya & Vlok, *Psoralea tenuifolia* L., and *Psoralea verrucosa* Willd.) (Stirton et al. 2012). Two new species also occurring on specialised substrates are reported from the Core Cape Region and are described below. *Psoralea diturnerae* is endemic to arid fynbos on the northern slopes of the Outeniqua mountains in the Camferskloof area, on acidic lithosol (Glenrosa and Mispah forms) sandstone of the Table Mountain group of soils (Rebelo et al. 2006) while *Psoralea vanberkelae* is endemic to the rocky quartzitic outcrops of the South Outeniqua Sandstone Fynbos vegetation at the Fynbos Private Nature Reserve (Robberg Coastal Corridor, Rooikrans).

All *Psoralea* species bearing (3)5–11(19)-foliolate leaves tend to be lumped together as the *Psoralea pinnata* species complex. This complex is thought to contain at least 28 species among which only 10 have been described formally. The complex includes all members of the genus *Psoralea* L. that fall within the broad concept of *Psoralea pinnata* as described by Linnaeus (1753). The major features of Linnaeus’ concept of *Psoralea pinnata* are: arborescent or shrubby, densely branched, pubescent or glabrous, leaves imparipinnate, in 3−5 pairs, linear or linear lanceolate, acute, very narrow, pedicels axillary, long or short, bracteolate (now cupulum) beyond the middle, calyx very variable in incision and pubescence. At the time of Linnaeus’ description, only 5 species were recognised. However, several species were described later by various authors including de Candolle, Jacquin, Poiret and Harvey. The following two new species are part of this complex as expanded progressively by these authors. Our current phenetic analysis, results not reported here, shows that only 24 species can be recognise as members of this complex. Most of the undescribed species are known only by their informal names in herbaria, and in publications describing the plants of the region e.g., Manning and Godlblatt (2012). Some of these have been included as ‘sp. nov’ in the Red Data list of southern African plants (Raimondo et al. 2009). These undescribed species will be described in a separate paper.

In the descriptions below, reference is made of a cupulum; an unusual and unique structure in the Fabaceae. In *Psoralea* there are no free bracts or bracteoles on or below the calyx. Instead these are fused into a complex cupulate structure which may occur anywhere along the peduncle between its base and apex. The cupulum is a diagnostic feature of most species (Tucker and Stirton 1981).

## Species treatment

### Psoralea diturnerae

Taxon classificationPlantaeFabalesFabaceae

A. Bello, C.H. Stirt. & Muasya sp. nov.

urn:lsid:ipni.org:names:77144551-1

#### Note.

Similar to *Psoralea pinnata* L., but differs in being a resprouter with massed short shoots from a woody rootstock (versus much-branched reseeder with single stem in *Psoralea pinnata*); grooved 3-foliolate leaves (versus 7−9-foliolate); flowers 1−3 per axil (versus 1−6).

#### Type.

SOUTH AFRICA. Western Cape, Oudtshoorn (−3322), Northern foothills of Outeniqua mountains, Camferskloof (–CD), 33°50'42.20"S, 22°25'8.93"E, 14 February 2014, *A. Bello, A.M. Muasya, & C.H. Stirton 41* (holotype: BOL!; isotypes: K!, NBG!, PRE!).

#### Description.

**Figure 1.**
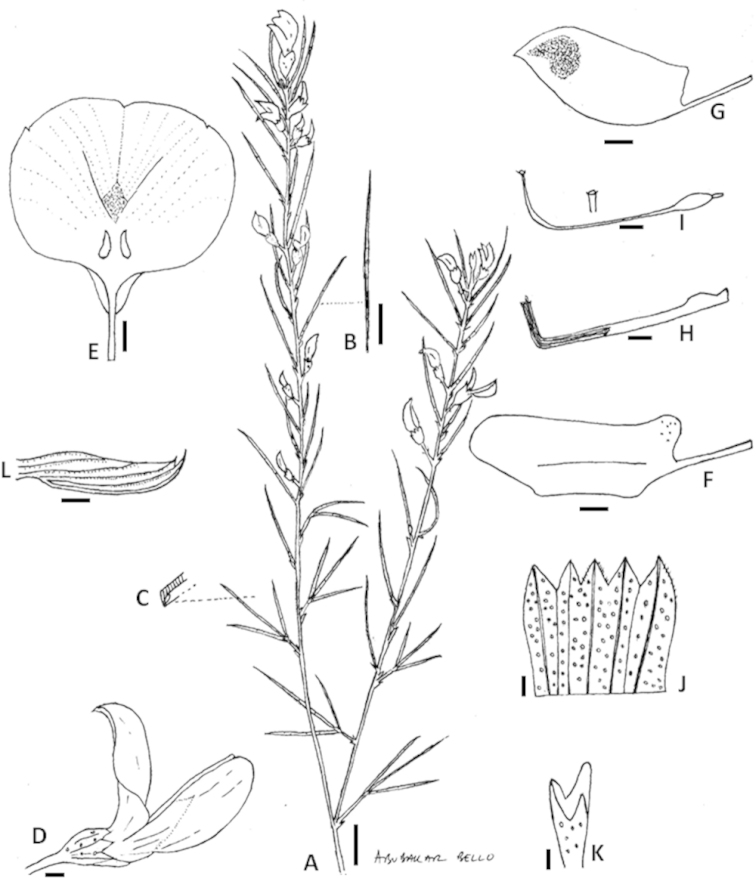
*Psoralea diturnerae* A. Bello, C.H. Stirt. & Muasya **A** flowering branch **B** leaf **C** transverse section of the leaflets **D** flower side view **E** standard petal **F** wing petal **G** keel petal **H** androecium **I** gynoecium showing the stigma **J** outer surface of calyx opened out **K** trifid cupulum **L** bud. Scale bars: **A, B**=1 cm; **D–K**=1 mm. Line drawing by Abubakar Bello from voucher *A. Bello, C.H. Stirton & A.M. Muasya 41* (BOL).

**Plate 1.**
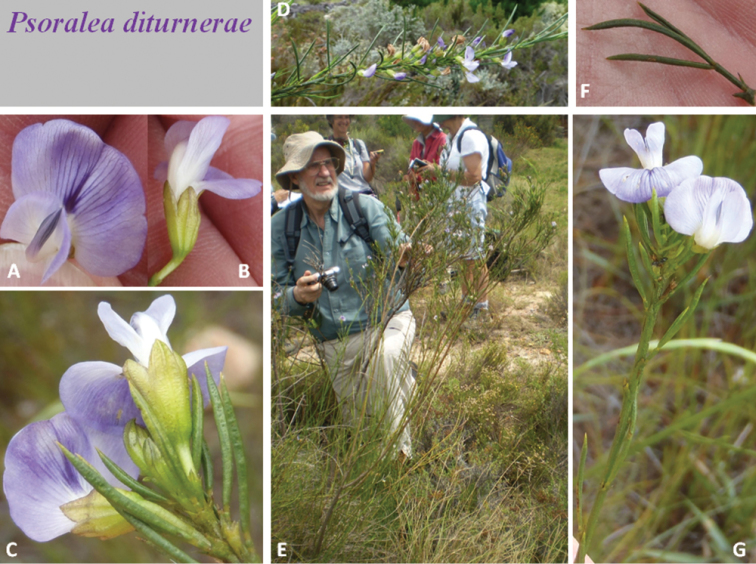
*Psoralea diturnerae* A. Bello, C.H. Stirt. & Muasya **A** front view of flower **B** side view of flower **C** apex of flowering shoot **D** seasonal flowering shoot **E** habit with C.H. Stirton **F** leaf **G** short flowering shoot. Photographs Nicky van Berkel (**A−C, F & G**), Abubakar Bello (**D**) and Sandra Falanga (**E**). Voucher *A. Bello, A.M. Muasya & C.H. Stirton 41* (BOL).

*Habit* an erect shrub up to 2 m tall, resprouter. *Stems* 1–3, bare with bursts of seasonal shoots in upper parts, with wide internodes; brown, covered in white storied lenticels; young seasonal shoots green, glabrous, glandular; flowering shoots produced seasonally on old stems, leafy along their entire length; plants also produce numerous sterile “water” shoots up to 1 m tall giving the plant an untidy restioid appearance. *Leaves* 3-foliolate at the base of each seasonal shoot, reducing to 1-foliolate thereafter, glabrous; leaf size variable, larger on water shoots from the rootstock (30–45 mm long, 30–40 mm wide); petiole 2–3 mm long; terminal leaflet of flowering shoots longest (20–40 mm long), basal pair (25–35 mm long), all 1.0–1.3 mm wide; glabrous, dark green; grooved, apex acuminate, base rounded; stipules 2–3 mm long, fused for half their length to the petiole, rigid, triangular, semi-patent, those on water shoots are longer, green and arching, rapidly senescent on flowering shoots. *Inflorescences* axillary along the length of seasonal shoots; flowers 1−3 per axil; peduncles absent or <1 mm long, terminated by a tri-toothed cupulum; cupulum lower tooth longest, acuminate, upper two teeth fused for half their length, yellowish, rapidly senescent, 1.0–1.2 mm long; pedicels 1–2 mm long *Flowers* 10–12 mm long, mauve to purple and white, borne 1–3 in leaf axils along seasonal flowering shoots. *Calyx* 5–6 mm long, 4 mm wide, pale green; tube 4 mm long, glabrous, ribbed; teeth triangular, equal, shorter than the tube, 2 mm long, carinal tooth cucullate at apex; glandular, margins ciliate with black hairs, inner face of teeth densely black-haired. *Standard petal* broadly elliptic to broadly ovate, 9–10 mm long, 8–10 mm wide; claw 2–3 mm long; mauve to purple, nectar “guide” situated above the strongly developed free appendages above the apex of the claw and comprised of a basal white area from which emerges a trifid purple flash that bleeds off into purple veins. *Wing petals* 9–11 mm long, 4 mm wide; claw 3 mm long; locked into keel indentation but not fused with it; longer than the keel; petal sculpturing present, upper basal, comprising 7–8 transcostal parallel lamellae. *Keel* 6 mm long, 3 mm wide; claw 5 mm long; apex dark purple. *Androecium* 9 mm long; tenth stamen free; sheath split adaxially, fenestrate; nectarial ring present, 0.5 mm high. *Pistil* 9 mm long; ovary 1.5 mm long, stipitate, glabrous but sparsely covered near distal end in curved stalked glands; ovules 1; style thickened at point of flexure, height of curvature 2 mm; stigma erect, penicillate. *Fruits* unknown. *Seeds* unknown (Fig. 1, Plate 1).

#### Habitat.

This species occurs in a small area of arid fynbos on the acidic lithosol soils (Glenrosa and Mispah forms) of North Outeniqua Sandstone Fynbos vegetation (FFs 18; Rebelo et al. 2006). The plant grows along streams or near water in the valley bottom, but is also found higher up the lower slopes along seepages.

#### Flowering time.

December to February.

#### Altitude.

Known from 620–667 m.

#### Distribution.

*Psoralea diturnerae* is narrowly endemic to the northern slopes of the Outeniqua mountains in the Camferskloof area, George, Western Cape Province of South Africa (Fig. 3). Unlike other species of *Psoralea* (e.g. *Psoralea odoratissima* Jacq., *Psoralea pinnata* L., and *Psoralea speciosa* Eckl. & Zeyh.) that are colonial, this species is occasional in the landscape across a wider area.

#### Etymology.

The specific epithet *diturnerae* honours Ms. Di Turner, ispotter (http://www.ispot.org.za/user/10170), the leader of the Outramps group of the Custodians of Rare and Endangered Wild flowers (C.R.E.W., South Africa) and her merry band of walkers who brought this species to our attention and sent us reference material and photographs. Her energy and drive has made her group the most active C.R.E.W. group in South Africa.

#### Conservation status.

*Psoralea diturnerae* is very rare and has only been found in the Camferskloof area on the northern slopes of Outeniqua mountains. So far only eight live individuals have been recorded in its habitat which is in a privately protected area but under possible threat from the nearby alien pine plantations. It is, however, possible that other individuals will emerge after the next fire as the plants we saw were old and leggy. Further surveys are being planned to find more individuals. We therefore assess this species to be Vulnerable under the South African Red List categories and criteria (VU D2, von Staden et al. 2009, IUCN 3.1, 2012a, 2012b).

#### Discussion.

*Psoralea diturnerae* is a recent discovery and is part of the *Psoralea pinnata* complex. It is a suffrutex with a restioid appearance of massed short shoots arising from a woody rootstock and from which emerge 1–3 long shoots that branch in their upper parts. It also has grooved 3-foliolate clasping glabrous needle-like leaves with the terminal leaflet longest. It bears rigid, triangular, semi-patent, rapidly senescent stipules, 1–3-flowered axillary inflorescences produced for long lengths of the flowering shoot and a white to purple standard petal with a white trifid central flash above the strongly developed auricles. It can also be recognised by its glabrous pale green calyx with purple flushes. *Psoralea pinnata* by contrast is a much-branched reseeding shrub to small tree up to 5 m tall with 7–9-foliolate linear, villoso-pubescent spreading leaves with the terminal leaflet shortest; has subulate recurved persistent stipules that become woody when leaves are shed, pale mauve or pale blue flowers borne along flowering shoots in pseudo-inflorescences, hidden within the subtending leaves, and the yellowish green, white (mostly) and black-haired calyces (Table 1). The species are allopatric.

#### Additional specimens examined.

Camferskloof, northern slopes of the Outeniqua Mountains, 33°50'56.9"S, 22°24'54.7"E (3322CD), Sandstone Fynbos, 622 m, 14 February 2014, *A. Bello, C.H. Stirton & A.M. Muasya 43* (BOL).

Camferskloof, northern slopes of the Outeniqua Mountains, 33°51'07.2"S, 22°25'04.7"E (3322CD), Sandstone Fynbos, 667 m, 23 January 2013, *Nicky van Berkel 1120* (BOL).

### Psoralea vanberkelae

Taxon classificationPlantaeFabalesFabaceae

C.H. Stirt., A. Bello & Muasya sp. nov.

urn:lsid:ipni.org:names:77144552-1

#### Note.

Similar to *Psoralea pinnata* L., but differs in being a short (less than 1 m) resprouter with sprawling and mounding habit (versus tall reseeder to 5 m with single stem in *Psoralea pinnata*); short 5-foliolate leaves (versus 7−9-foliolate); flowers solitary per axil (versus 1−6).

#### Type.

SOUTH AFRICA. Western Cape, Knysna (−3423), Robberg Coastal Corridor, Fynbos Private Nature Reserve (–AB), 34°05'51.72"S, 23°17'5.82"E, 134 m, 18 October 2013, *N. van Berkel 1118* (holotype: BOL!; isotypes: GRA!, K!, NBG!, SCHG!, PRE!).

#### Description.

**Figure 2.**
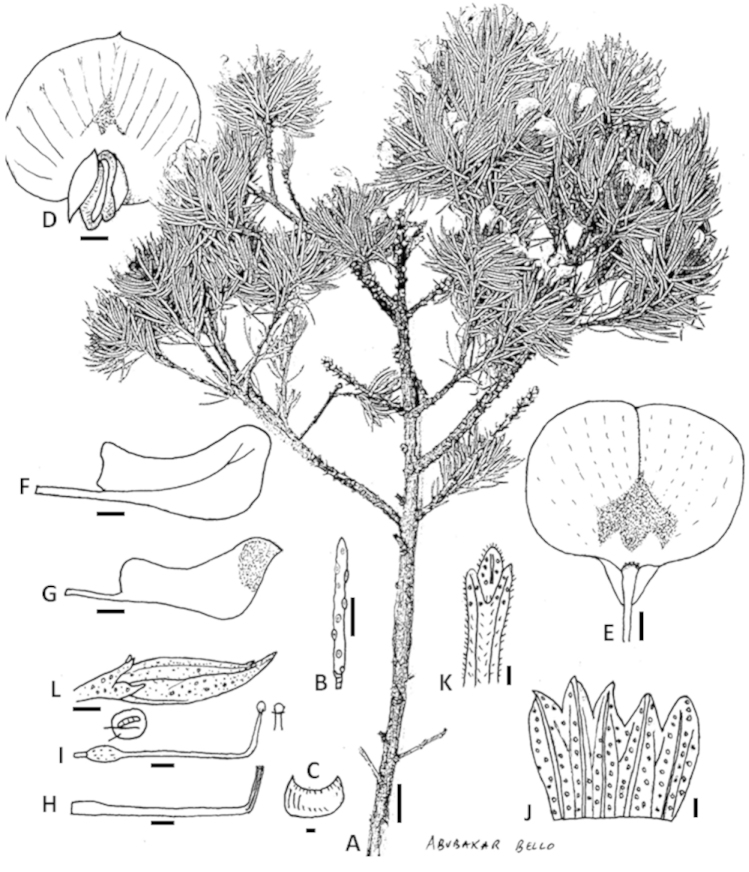
*Psoralea vanberkelae* C.H. Stirt., A. Bello & Muasya **A** flowering branch **B** leaf **C** stipule **D** flower viewed from the front **E** standard petal showing the M-shaped nectar patch **F** wing petal **G** keel petal **H** androecium **I** gynoecium showing the stigma **J** outer surface of calyx opened out **K** trifid cupulum **L** bud. Scale bars: **A, B**=1 cm; **C–L**=1 mm. Line drawing by Abubakar Bello from voucher *N. van Berkel 1118* (BOL).

**Plate 2.**
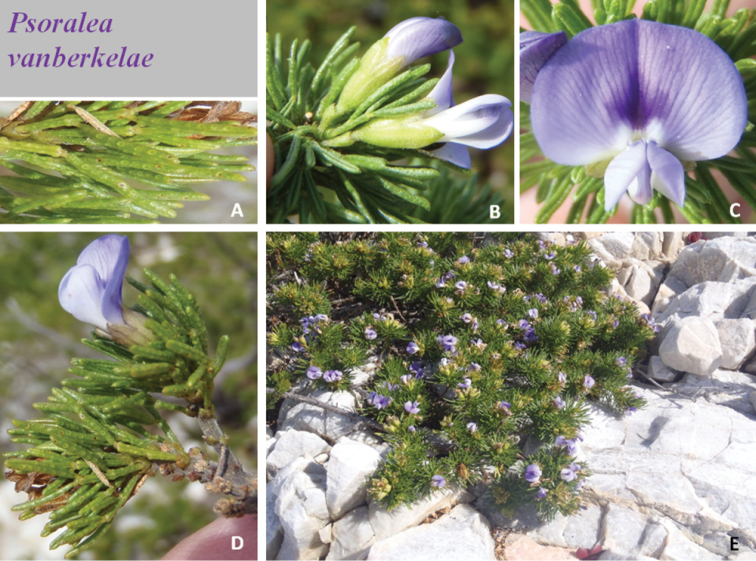
*Psoralea vanberkelae* C.H. Stirt., A. Bello & Muasya **A** details of glands on leaflets **B** base view of flowers showing cupulums below calyces **C** flower **D** short seasonal shoot with flower **E** habit. Photographs Nicky van Berkel. Voucher *N. van Berkel 1118* (BOL).

*Habit* a small sprawling and mounding shrub to 60 cm tall and up to 1.5 m wide, resprouter. *Stems* 1–10, branching in upper parts of stems; branches erect, rough, grey, mostly bare except for upper parts; young seasonal shoots rough, blackish, hairy. *Leaves* 9 mm long, 10 mm wide, pinnately 5-foliolate, linear oblong, petiolate, fleshy, basal leaves of seasonal shoots smallest, patent to semi-erect, surface bumpy, glabrous; glands raised, hyaline but drying reddish brown to black, rachis grooved; basal leaflet pair 10 mm long, 0.5 mm wide, equal to or slightly shorter than terminal leaflet; terminal leaflet 10−11 mm long, 0.5 mm wide, flat on adaxial surface with a distinct furrow; stipules 2 mm long, 1 mm wide, straight, fused, joined by a bridge of tissue, glabrescent, teeth broadly triangular, apex acute, fleshy, persistent, becoming prominent and woody when leaves are shed, hairy, hairs short and stubby, covered densely with large raised glands. *Inflorescences* axillary in upper nodes of short seasonal shoots; peduncle short, 2 mm long, hairy; peduncle bracts paired, minute; cupulum 1 mm long, pale green, trifid, shortly triangular, lobes equal, black-haired, covered in large glands, drying reddish brown; pedicel 2 mm long. *Flowers* 10−11 mm long, pale to intense mauve to blue, borne solitary per axil. *Calyx* 6 mm long, 4 mm wide; tube 4 mm long, ribbed; teeth equal, shorter than tube, 2 mm long, pale green, sparsely covered in small black flat hairs and densely encrusted with mixed sized glands on outside; margins of teeth densely black ciliate, inside of teeth densely stubby black-haired; vexillar teeth scarcely fused above tube. *Standard petal* 9−10 mm long, 7−8 mm wide; claw 2−3 mm long, flattened, erect; very broadly ovate, reflexed to 90 degrees, apex rounded; mauve but dark purple in central area above the M-shaped white nectar “guide”, venation purple; callosities above the claw absent. *Wing petals* 6−7 mm long, 3−4 mm wide; claw 4−5 mm long; longer than keel petals, strongly folded once along middle, slightly billowy near apex, held parallel to keel, strongly auriculate; sculpturing present, upper basal comprised of 4−5 transcostal lamellae. *Keel* 5−6 mm long, 3−4 mm wide; claw up to 5 mm long. *Androecium* 7 mm long; tenth stamen free; sheath split abaxially, fenestrate; nectarial ring present, 0.3 mm high. *Pistil* 7 mm long; ovary 2 mm long, stipitate, glabrous but sparsely covered in curved stalked glands across sides; ovules 1; stigma penicillate. *Fruits* 1, 5 mm long, 3 mm wide, papery, rugose, reticulate, brown. *Seeds* 4 mm long, 2.5 mm wide, oblong-elliptic, khaki with black mottles and flecks, hilum central (Fig. 2, Plate 2).

#### Habitat.

Endemic to South Outeniqua Sandstone Fynbos (FFs19, Rebelo et al. 2006). The vegetation type is a mixture of Eastern Fynbos and Renosterveld. It grows in full sun on sandy soils over Peninsula formation quartzite on a gentle slope. The area was subjected to a controlled burn in April 2008, so plants are becoming old and starting to die back.

#### Flowering time.

August to November.

#### Altitude.

70−150 m.

#### Distribution.

**Figure 3.**
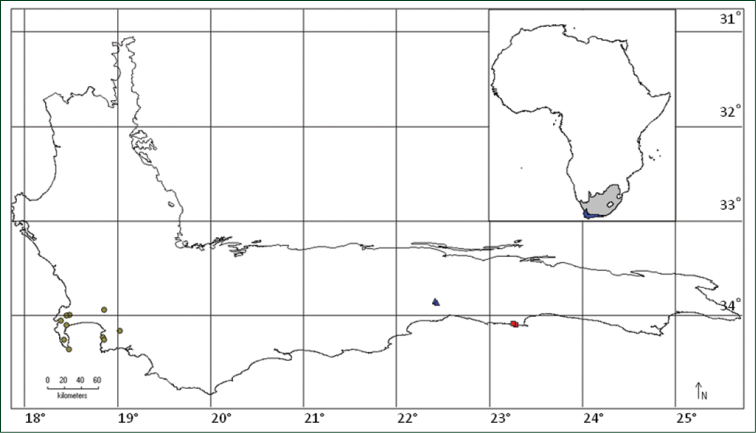
Distribution of *Psoralea pinnata* (circles), *Psoralea diturnerae* (triangles) and *Psoralea vanberkelae* (squares). The top right map of Africa shows the position of the Core Cape Region (blue) in South Africa (grey).

*Psoralea vanberkelae* is a narrow endemic. It is known from some hundreds of individuals in an area of 500 × 500 m along the George to Knysna coastal stretch of the Indian Ocean and also from Cairnbrogie (Nicky van Berkel pers. comm., photographs) all in Western Cape Province of South Africa (Fig. 3).

#### Etymology.

The specific epithet *vanberkelae* honours Ms. Nicky van Berkel, a C.R.E.W. volunteer and iSpotter (“Nicky”; http://www.ispot.org.za/user/10095), who brought this species to our attention and sent us reference material and photographs. Like many plant enthusiasts from C.R.E.W. she plays a valuable role in establishing the conservation status of plants in her area. The plant is a beautiful flagship species for a very threatened habitat.

#### Conservation status.

*Psoralea vanberkelae* is locally abundant in its habitat and the main population is protected by private ownership (Fynbos Private Nature Reserve). It is, however, restricted by a narrow range of distribution (area less than 20 km^2^). The coastal stretches where the plants occur are all on private land with limited access. The cliff edges rise sharply from the sea and their escarpments are not easy to access. We therefore assess this species to be Vulnerable under the South African Red List categories and criteria (VU D2, von Staden et al. 2009, IUCN 3.1, 2012a, 2012b).

#### Discussion.

*Psoralea vanberkelae* is a recent discovery and is part of the *Psoralea pinnata* complex. It is a small, colonial, resprouting, sprawling and mounding shrub to 60 cm tall and up to 1.5 m wide. It has clasping, tightly packed, 10–11 mm long leaves on short shoots. It also has glabrous leaflets with large round impressed glands. Its terminal leaflet is in most cases longest. It has pale to intense mauve to blue flowers borne at the end of short flowering shoots in pseudo-inflorescences and held above the subtending leaves. *Psoralea pinnata* on the other hand is a taller much-branched reseeding shrub to small tree up to 5 m tall with 7–9-foliolate linear, 20–45 mm long, villoso-pubescent spreading leaves with the terminal leaflet shortest (Table 1).

#### Additional specimens examined.

Robberg Coastal Corridor, Fynbos Private Nature Reserve Section, Knysna, 34°05'53.84"S, 23°17'4.56"E (3423AB), South Outeniqua Sandstone Fynbos, 134 m, 14 February 2014, *A. Bello, C.H. Stirton & A.M. Muasya 53* (BOL).

**Table 1.** Some diagnostic characters distinguishing the two new species from *Psoralea pinnata*.

S/No	Characteristics	*Psoralea pinnata*	*Psoralea diturnerae*	*Psoralea vanberkelae*
1	Habit	tall shrub to 5 m	small shrub to 2 m	shrub, less than 1 m
2	Regeneration strategy	reseeder	resprouter	resprouter
3	Appearance	erect, multi-branched in upper parts	erect, less branched in upper parts with numerous sterile short basal shoots	sprawling and mounding, multi-branched
4	Leaves	7−9-foliolate, 20–45 mm long	1−3-foliolate, 30–45 mm long	5-foliolate, 9–11 mm long
5	Inflorescence	flowers 1−6 per axil, pale mauve or pale blue, 14−18 mm long, hidden within the subtending leaflets	flowers 1−3 per axil, mauve to purple and white, 10−12 mm long, exposed within the subtending leaflets	flowers solitary per axil, pale to intense mauve to blue, 10−11 mm long, exposed above the subtending leaflets
6	Calyx	8−9 mm long, yellowish green	5−6 mm long, pale green	6 mm long, pale green
7	Cupulum	trifid, lobes equal, overlapping the calyx	trifid, lobes unequal, free from the calyx	trifid, lobes equal, free from the calyx

## Supplementary Material

XML Treatment for Psoralea diturnerae

XML Treatment for Psoralea vanberkelae

## References

[B1] Dludlu MN Stirton CH Chimphango SBM Bello A Muasya AM (2013) Phylogenetic position of the southern African members of the tribe Psoraleeae based on molecular and morphological data. South African Journal of Botany 89: 150−155. doi: 10.1016/j.sajb.2013.06.019

[B2] IUCN (2012a) Guidelines for application of IUCN Red List Criteria at Regional and National Levels: Version 4.0. IUCN, Gland, Switzerland and Cambridge.

[B3] IUCN (2012b) IUCN Red list Categories and Criteria: Version 3.1 (second ed.). IUCN, Gland, Switzerland and Cambridge.

[B4] Linnaeus C (1753) Species plantarum. Facsimile of edition 1. Bernard Quariten Ltd., London.

[B5] Rebelo AG Boucher C Helme N Mucina L Rutherford MC (2006) In: Mucina L Rutherford MC (Eds) Vegetation map of South Africa, Lesotho and Swaziland, Strelitzia 19. South African National Biodiversity Institute, Pretoria.

[B6] Stirton CH Schutte AL (2012) *Psoralea*. In: Manning J Goldblatt P (Eds) Plants of the Greater Cape Floristic Region 1: the Core Cape Flora, Strelitzia 29. South African National Biodiversity Institute, Pretoria, 571−575.

[B7] Stirton CH Schutte AL (2000) *Psoralea*. In: Goldblatt P Manning JC (Eds) Cape Plants: a Conspectus of the Cape Flora of South Africa. National Botanical Institute of South Africa, Pretoria, 505–507.

[B8] Stirton CH Clark RV Barker NP Muasya AM (2011) *Psoralea margaretiflora* (Psoraleeae, Fabaceae): A new species from the Sneeuberg Centre of Floristic Endemism, Eastern Cape, South Africa. Phytokeys 5: 31–38. doi: 10.3897/phytokeys.5.1585 22171191 10.3897/phytokeys.5.1585 PMC3174448

[B9] Stirton CH Muasya AM Vlok J (2012) *Psoralea karooensis* (Psoraleeae, Fabaceae), a new species from the Klein Karoo region of South Africa. Phytokeys 17: 19–23. doi: 10.3897/phytokeys.17.3672 23233814 10.3897/phytokeys.17.3672 PMC3519350

[B10] Tucker SC Stirton CH (1990) Development of the cymose inflorescence, cupulum and flower of *Psoralea pinnata* (Leguminosae: Papilionoideae: Psoraleeae). Botanical Journal of the Linnean Society 106: 209–227. doi: 10.1111/j.1095-8339.1991.tb02292.x

[B11] von Staden L Raimondo D Foden W (2009) Approach to Red List Assessments. In: Raimondo D von Staden L Foden W Victor JE Helme NA Turner RC Kamundi DA Manyama PA (Eds) Red List of South African Plants.South African National Biodiversity Institute, Pretoria, 6–18.

